# The combination of non-contrast abbreviated MRI and alpha foetoprotein has high performance for hepatocellular carcinoma screening

**DOI:** 10.1007/s00330-023-09906-4

**Published:** 2023-07-18

**Authors:** Raphaël Girardet, Margaux Dubois, Gibran Manasseh, Mario Jreige, Céline Du Pasquier, Emma Canniff, Marianna Gulizia, Melissa Bonvin, Yasser Aleman, Bachir Taouli, Montserrat Fraga, Clarisse Dromain, Naik Vietti Violi

**Affiliations:** 1 Department of Radiology and Interventional Radiology, Lausanne University Hospital and Lausanne University, Lausanne, Switzerland; 2 Department of Gastro-enterology, Lausanne University Hospital and Lausanne University, Lausanne, Switzerland; 3 Biomedical Engineering and Imaging Institute, Icahn School of Medicine at Mount Sinai, New York, NY USA

**Keywords:** Magnetic resonance imaging, Carcinoma, hepatocellular, Contrast agent, Screening

## Abstract

**Objectives:**

This study aimed to compare two abbreviated MRI (AMRI) protocols to complete MRI for HCC detection: non-contrast (NC)-AMRI without/with alpha foetoprotein (AFP) and dynamic contrast-enhanced (Dyn)-AMRI.

**Methods:**

This retrospective single-center study included 351 patients (M/F: 264/87, mean age: 57y) with chronic liver disease, who underwent MRI for HCC surveillance between 2014 and 2020. Two reconstructed AMRI sets were obtained based on complete MRI: NC-AMRI (T2-weighted imaging (WI) + diffusion-WI) and Dyn-AMRI (T2-WI + dynamic T1-WI) and were assessed by 2 radiologists who reported all suspicious lesions, using LI-RADS/adapted LI-RADS classification. The reference standard was based on all available patient data. Inter-reader agreement was assessed and MRI diagnostic performance was compared to the reference standard.

**Results:**

The reference standard demonstrated 83/351 HCC-positive patients (prevalence: 23.6%, median size: 22 mm, and positive MRIs: 83/631). Inter-reader agreement was substantial for all sets. Sensitivities of Dyn-AMRI and complete MRI (both 92.8%) were similar, higher than NC-AMRI (72.3%, *p* < 0.001). Specificities were not different between sets. NC-AMRI + AFP (92.8%) had similar sensitivity to Dyn-AMRI and complete MRI. In patients with small size HCCs (≤ 2 cm), sensitivities of Dyn-AMRI (85.3%) and complete MRI (88.2%) remained similar (*p* = 0.564), also outperforming NC-AMRI (52.9%, *p* < 0.05). NC-AMRI + AFP had similar sensitivity (88.2%) to Dyn-AMRI and complete MRI (*p* = 0.706 and *p* = 1, respectively).

**Conclusions:**

Dyn-AMRI has similar diagnostic performance to complete MRI for HCC detection, while both outperform NC-AMRI, especially for small size HCCs. NC-AMRI + AFP demonstrates similar sensitivity to Dyn-AMRI and complete MRI.

**Clinical relevance statement:**

Due to the low sensitivity of ultrasound for hepatocellular screening, new screening methods are needed. Abbreviated MRI (AMRI) is a candidate, especially non-contrast AMRI with serum alpha foetoprotein as the acquisition time is low, without the need for contrast medium injection.

**Key Points:**

*• Dynamic contrast-enhanced abbreviated MRI using extracellular gadolinium-based contrast agent and complete MRI have similar diagnostic performance for hepatocellular carcinoma detection in an at-risk population.*

*• Non-contrast abbreviated MRI with alpha foetoprotein has similar diagnostic performance to dynamic contrast-enhanced abbreviated MRI and complete MRI, including when considering small size hepatocellular carcinoma  ≤ 2 cm.*

*• Non-contrast abbreviated MRI and dynamic contrast-enhanced abbreviated MRI can be performed in 7 and 10 min, excluding patient setup time.*

**Supplementary information:**

The online version contains supplementary material available at 10.1007/s00330-023-09906-4.

## Introduction

Cirrhosis is increasing worldwide and is a major cause of death, partly related to the increased risk of hepatocellular carcinoma (HCC) in these patients 1. Clinical practice guidelines recommend bi-annual screening with abdominal ultrasound (US) with or without serum alpha foetoprotein (AFP), in patients at risk of HCC [2–4]. However, US demonstrates limited detection sensitivity for early HCC (47%), particularly in patients with large body habitus and/or advanced cirrhosis [5, 6]. HCC size at time of diagnosis is crucial, as this is an important factor for management decision and prognosis [2].

The value of AFP in HCC surveillance is debated, as a meta-analysis including 13 prospective studies found no added value of AFP [7]. However, four subsequent prospective studies [5, 8–10] and one meta-analysis [6] showed an added value for AFP. As a consequence, the most recent American Association for the Study of Liver Diseases (AASLD), European and Asia-Pacific guidelines recommend using US with or without AFP for HCC surveillance [2–4].

Although MRI is the imaging method of reference for HCC diagnosis and staging, current practice guidelines do not advocate the use of MRI for HCC surveillance, due to long exam duration, limited access, and costs [2, 11, 12]. In the daily practice, MRI is often used in transplant centers for HCC surveillance [13].

Abbreviated MRI (AMRI) protocols are being evaluated as an alternative to US for HCC surveillance, relying on the use of a few selected MRI sequences. The main goal of AMRI is to keep acceptable diagnostic performance for HCC detection while reducing acquisition time and cost [14].

Several studies have reported various AMRI protocols, including non-contrast AMRI (NC-AMRI) [15–20], hepato-biliary phase AMRI (HBP-AMRI) using hepato-specific contrast agent (Primovist/Eovist, Bayer Healthcare) [15, 19–24], and dynamic contrast-enhanced AMRI (Dyn-AMRI) using hepato-specific contrast agents or extracellular contrast agents (ECCA) [19, 25–27]. A Korean prospective study comparing US with NC-AMRI evidenced per exam sensitivity of 27.9% and 79.1%, respectively, demonstrating the potential added value of AMRI in the context of HCC surveillance [18]. While NC-AMRI and HBP-AMRI have been assessed, data are still scarce regarding HCC detection with Dyn-AMRI [19, 25, 27]. Moreover, the addition of AFP to AMRI has not been evaluated so far.

Our primary objective was to evaluate the performance of NC-AMRI + / − AFP and Dyn-AMRI compared to a complete MRI protocol for HCC detection in a population at risk.

## Materials and methods

### Study design

This retrospective single-center study was approved by the local Institutional Review Board (ID CER-VD 2020-00680), which waived the need for signed informed consent.

**Fig. 1:**
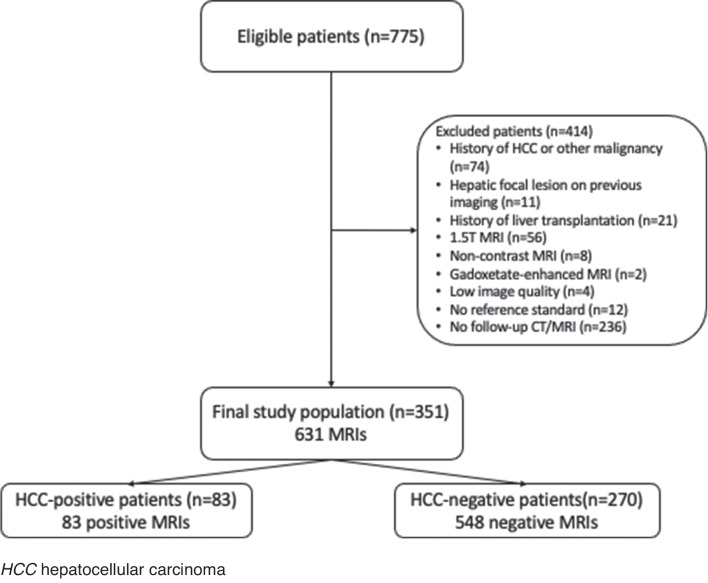
Flowchart of patient selection

Eligible patients were identified using our electronic imaging database that was queried for patients with a liver MRI performed for HCC surveillance between 2014 and 2020. MRIs performed for HCC screening were available, as hepatologists at our institution request MRI in alternation with US every 6 months. Study inclusion criteria were as follows: (1) adult patients (> 18 y.o); (2) patients at risk for HCC according to the European Association for the Study of the Liver (EASL) clinical guidelines (cirrhosis or advanced fibrosis (METAVIR score F3 or higher) and/or chronic hepatitis) [2]; (3) MRI performed on a 3-T machine. Exclusion criteria are listed in Fig. 1.

A maximum of 3 MRIs per patient were included, even if more were performed between 2014 and 2020. For HCC-positive patients, further MRIs after diagnosis were not included. Among 775 patients, 414 were excluded. A total of 351 patients were included, with 631 MRIs.

### Data collection

All patients included in the present study had regular follow-up as outpatient at the liver clinic of our institution and were retrospectively characterized using our clinical workflow software (Soarian, Siemens Medical Solutions). The study coordinator (R.G.) collected demographic, clinical, and biological data.

### MRI acquisition and extraction of AMRI sets

All liver MRIs were performed on two different 3-T MRI systems; Magnetom Prisma and Skyra (Siemens Healthineers), using a standard liver dedicated protocol. Details on the imaging parameters are provided in Supplementary Material. 

**Fig. 2:**
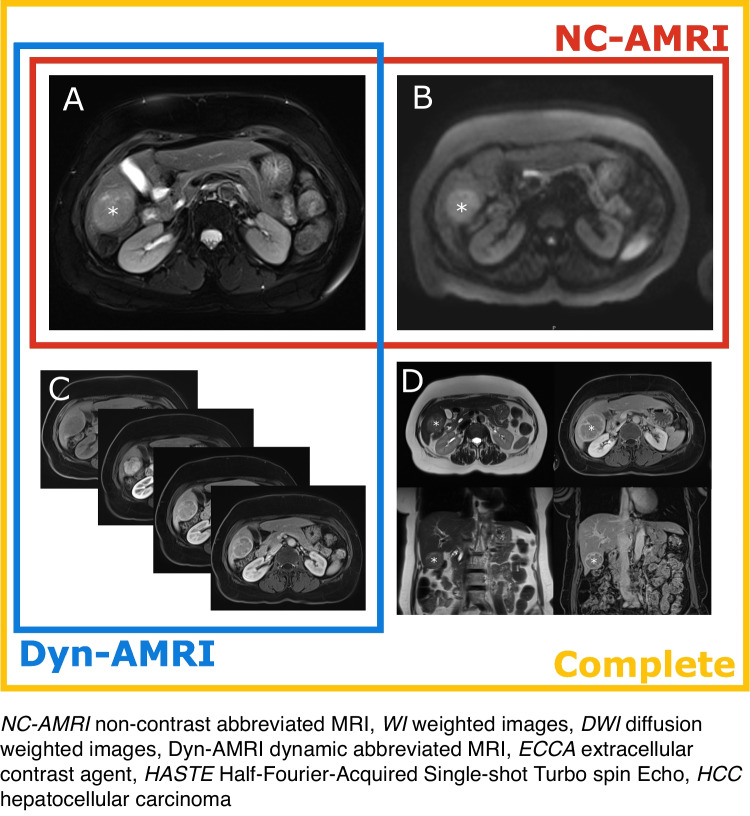
Sequences included in our two AMRI protocols (NC-AMRI, Dyn-AMRI) and complete MRI. NC-AMRI (red square) included fat-saturated T2WI (**A**) and DWI (**B**). Dyn-AMRI (blue square) included fat-saturated T2WI (**A**), native T1WI and dynamic ECCA-enhanced T1WI arterial, portal and transitional phase (**C**), without delayed axial and coronal 5 min phase. Complete MRI (yellow square) included fat-saturated T2WI (**A**), axial and coronal T2WI HASTE (**D**), DWI (**B**), native T1WI and dynamic ECCA-enhanced T1WI arterial, portal, transitional (**C**) and delayed axial and coronal 5 min phase (**D**). To note, a LI-RADS 5 biopsy-proven HCC of segment VI is visible on all sequences (asterisks)

Two AMRI protocols were extracted from the complete MRI and assessed separately (Fig. 2): (1) NC-AMRI, including axial fat-suppressed T2-weighted imaging (WI) + diffusion-weighted imaging (DWI), and (2) Dyn-AMRI, including axial fat-suppressed T2WI + axial dynamic contrast-enhanced T1WI (unenhanced, arterial, portal venous, and transitional phase at 3 min). Axial fat-suppressed T2WI was included on all sets, to improve lesion characterization (especially for cysts and hemangiomas).

The average estimated acquisition times based on our clinical practice (without setup time) are 7 min for NC-AMRI, 10 min for Dyn-AMRI, and 26 min for complete MRI.

### Image analysis

Two independent radiologists (G.M. and C.D., with 2 and 24 years of experience in abdominal imaging, respectively) analyzed the 2 AMRI protocols and the complete MRI on a Picture Archiving and Communication System (Siemens Healthineers). Each reader reviewed half of each set, plus 100 common cases for inter-reader agreement analysis, in two sessions separated by at least 6 weeks to reduce recall bias. In one session, NC-AMRI was assessed, immediately followed by complete MRI. In the other session, Dyn-AMRI was assessed. MRIs were reviewed in random order, without access to previous examinations. Up to 5 observations per MRI were selected based on the largest size (excluding typical cysts). For each selected observation, readers recorded the observation size and location.

In the NC-AMRI, each lesion was scored using a scoring system adapted from the US LI-RADS [28]: negative (no lesion or clearly benign), subthreshold (< 10 mm and not clearly benign), positive (≥ 10 mm and hypersignal not attributable to cirrhosis or benign lesions). The Dyn-AMRI and complete MRI sets were scored using Liver Reporting and Data System (LI-RADS) 2018 [29].

An MRI was considered positive on NC-AMRI when at least 1 lesion was scored positive, or on Dyn-AMRI/complete MRI when at least one lesion was scored LI-RADS 4, 5, Tumor in vein (TIV) or M, as previously categorized [25]. All other cases were considered negative. As readers did not have access to previous imaging, threshold growth was not included in the assessment.

A combination between NC-AMRI and AFP was performed. AFP value was considered positive when  > 5 kUI/l, according to our institutional guidelines. The patient was considered HCC-positive when either NC-AMRI and/or AFP was positive.

### Reference standard

Each MRI was classified as positive/negative for HCC by the study coordinator (R.G., radiologist with 2 years of experience in abdominal imaging), using all patient data available including imaging examinations, pathology when available, multidisciplinary tumor board decision, and subsequent treatment. On imaging, patients with LI-RADS 5 and TIV lesions were considered HCC-positive. Patients with observations scored LI-RADS 4 were either initially biopsied and considered positive versus negative, or considered negative when not biopsied and stable at 6 months. Patients with LI-RADS M lesions were all biopsied, and all were HCC in this study. MRIs with lesions assessed LI-RADS 3 on reference standard with negative biopsy or without threshold growth on subsequent CT or MRI follow-up were considered negative. MRIs were considered negative when no lesion was found, or when all observations were scored LI-RADS 1 or 2. For MRIs considered negative, a subsequent CT or MRI follow-up (with a delay of at least 6 months) was required to confirm the negative result.

### Statistical analysis

Kappa coefficient (*K*) was used to evaluate inter-reader agreement. The level of agreement was interpreted as poor (*K* < 0), slight (0 ≤ *K* ≤ 0.2), fair (0.2 < *K* ≤ 0.4), moderate (0.4 < *K* ≤ 0.6), substantial (0.6 < *K* ≤ 0.8), or almost perfect (*K* > 0.8). The diagnostic performance of both AMRI protocols and complete MRI with or without AFP for NC-AMRI was compared to the reference standard. The confusion matrices were computed and sensitivity and specificity were calculated. Comparisons of sensitivity and specificity between reading sets and the reference standard were based on the McNemar test. Receiver operating characteristic (ROC) curves were analyzed, with computation of area under the ROC curve (ROC area). ROC areas were compared using the nonparametric chi-squared test of equality. Identical analysis was performed on a selected subgroup of patients with small size HCCs (≤ 2 cm). All statistical tests were conducted at the two-sided 5% significance level. The Statistics and Machine Learning Toolbox (v12.0) included in Matlab (v2020b) and STATA, version 14.2 (STATA Corp.) were used to perform the analyses.

## Results

### Patient population

The final study population included 351 patients (M/F 264/87, median age 58 y.o, range: 19–83) with 631 MR examinations. Patient demographics and clinical characteristics are summarized in Table 1.

**Table 1:** Baseline patient characteristics

Characteristic	Study cohort (*n* = 351)	Patients with HCC (*n* = 83)	*p*
Sex (M/F)	264/87	62/21	1
Age (median, range)	58, 19–83	61, 34–81	< 0.001
Ethnicity			0.57
Caucasian	296 (84.3%)	72 (86.8%)	0.63
Asian	25 (7.1%)	5 (6%)	0.72
African	24 (6.9%)	2 (2.4%)	0.14
South American	6 (1.7%)	4 (4.8%)	0.09
Liver disease etiology			0.25
Alcohol consumption	180 (51.3%)	45 (54.2%)	
HCV	70 (20%)	25 (30.1%)	
HBV	42 (12%)	3 (3.6%)	
MAFLD	37 (10.5%)	7 (8.5%)	
Other*	18 (5.1%)	2 (2.4%)	
Unknown	4 (1.1%)	1 (1.2%)	
Cirrhosis			0.003
Yes	310 (88.3%)	81 (97.6%)	
No	41 (11.7%)	2 (2.4%)	
Child–Pugh class			0.22
A	234 (75.5%)	57 (70.4%)	0.13
B	56 (18.1%)	20 (24.7%)	0.7
C	18 (5.8%)	4 (4.9%)	0.06
Unknown	2 (0.6%)	0 (0%)	
HCC size (median, range)	-	22 mm (10–142 mm)	

^*^Autoimmune hepatitis, primary biliary cirrhosis, primary sclerosing cholangitis, hemochromatosis, alpha-1-antitrypsin deficiency

Abbreviations: *HBV* hepatitis B virus, *HCV* hepatitis C virus, *MAFLD* metabolic-associated fatty liver disease, ECOG Eastern Cooperative Oncology Group, *HCC* hepatocellular carcinoma

The majority of patients were cirrhotic (88.3%), Caucasian (84.3%) with a Child-Turcott-Pugh score A (75.5%). The predominant cause of cirrhosis was alcoholic liver disease (51.3%). Eighty-three patients (23.6%) had HCC according to the reference standard. Fifty HCCs were discovered on the first MR examination, 12 on the second MR examination, and 21 on the third MR examination. The median tumor size was 22 mm (standard deviation 20 mm, range 10–142 mm), considering the largest lesion for each patient. Forty-one patients had a single HCC lesion, while 13 patients had 2 or 3 lesions and 13 patients had 4 lesions or more. Thirty-four patients (41%) had small size HCC (≤ 2 cm). HCC and other observations found on MRI are detailed in Table 2.

**Table 2:** Description of liver observations in the study population, per patient

Observations	LI-RADS score	*n*
HCC		
	LI-RADS 5	66
	LI-RADS 4, biopsy-proven HCC	13
	LI-RADS M, biopsy-proven HCC	4
Benign		
Hemangioma	LI-RADS 1	3
Hepatocellular adenoma	LI-RADS 5	2
Previous abscess	LI-RADS 3	1
Echinococcosis	LI-RADS 2	1
Cirrhotic nodule on biopsy	LI-RADS 4	10
Presumed dysplastic nodule	LI-RADS 2 or 3, stable at 6 months	28
Dysplastic nodule on biopsy	LI-RADS 4	1
Indeterminate	LI-RADS 4, without biopsy, stable at 6 months	11

Abbreviations: *LI-RADS* Liver Reporting and Data System, *HCC* hepatocellular carcinoma

Compared to the whole cohort, patients with HCC were older (*p* < 0.001) and more likely to have cirrhosis (*p* = 0.003) (Table 1).

### HCC detection

#### Overall population

The inter-reader agreement for HCC detection was substantial for all reading sets (*K* range: 0.67–0.68).

The diagnostic performance of Dyn-AMRI (AUC 0.946) and complete MRI (0.944) was significantly higher than that of NC-AMRI (0.834, both *p* < 0.001), mainly due to higher sensitivity with Dyn-AMRI and complete MRI (both 92.8%) compared to NC-AMRI (72.3%, both *p* < 0.001) (Table 3). AUC, sensitivity, and specificity were similar between Dyn-AMRI and complete MRI (*p* = 0.906, 1 and 0.669, respectively).

**Table 3:** Diagnostic performance

Reading set	Sensitivity (ratio, 95% CI)	Specificity (ratio, 95% CI)	AUC (95% CI)
AFP (> 5 kUI/l)	63.9% (53/83, 52.6–74.1%)	98.5% (540/548, 97.1–99.4%)	0.812 (0.759–0.864)
NC-AMRI	72.3% (60/83, 61.4–81.6%)	94.5% (518/548, 92.3–96.3%)	0.834 (0.785–0.883)
Dyn-AMRI	92.8% (77/83, 84.9–97.3%)	96.3% (528/548, 94.4–97.8%)	0.946 (0.917–0.975)
Complete MRI	92.8% (77/83, 84.9–97.3%)	96% (526/548, 94–97.3%)	0.944 (0.915–0.973)
NC-AMRI + AFP	92.8% (77/83, 84.9–97.3%)	93.6% (513/548, 91.2–95.5%)	0.932 (0.902–0.962)
*p* values			
AFP vs NC-AMRI + AFP	< 0.001	< 0.001	< 0.001
NC-AMRI vs Dyn-AMRI	< 0.001	0.086	< 0.001
NC-AMRI vs complete MRI	< 0.001	0.102	< 0.001
Dyn-AMRI vs complete MRI	1	0.669	0.906
NC-AMRI + AFP vs NC-AMRI	< 0.001	0.025	< 0.001
NC-AMRI + AFP vs Dyn-AMRI	1	0.013	0.493
NC-AMRI + AFP vs complete MRI	1	0.012	0.548

Abbreviations: *CI* confidence intervals, *AUC* area under the curve, *AFP* alpha foetoprotein, *NC-AMRI* non-contrast abbreviated MRI, *Dyn-AMRI* dynamic abbreviated MRI

#### AFP combination to NC-AMRI

In the overall population, the combination of NC-AMRI + AFP significantly improved sensitivity (92.8%) compared to NC-AMRI alone (72.3%), thus 20.5% sensitivity improvement (*p* < 0.001). The diagnostic performance of NC-AMRI + AFP (AUC 0.932) was significantly higher compared to NC-AMRI alone (AUC 0.834, *p* < 0.001), whereas the specificity was significantly lower (93.6% versus 94.5%, *p* = 0.025).

NC-AMRI + AFP had similar sensitivity to Dyn-AMRI and complete MRI (all 92.8%, *p* = 1).

AFP alone had a sensitivity of 63.9%.

#### Small size HCCs (≤ 2 cm)

In the subgroup of patients with small size HCCs, the diagnostic performance and sensitivity of Dyn-AMRI (AUC 0.908, sensitivity 85.3%) and complete MRI (0.921, 88.2%) remained higher than those of NC-AMRI (0.737, 52.9%, all *p* < 0.05) (Table 4). The sensitivity of Dyn-AMRI, complete MRI, and NC-AMRI dropped by 7.5%, 4.6%, and 19.4%, respectively, compared to the overall population. AUC, sensitivity, and specificity were not significantly different between Dyn-AMRI and complete MRI (*p* = 0.621, 0.564, and 0.670, respectively).

**Table 4:** Small size HCCs (≤ 2 cm) diagnostic performance

Reading set	Sensitivity (ratio, 95% CI)	Specificity (ratio, 95% CI)	AUC (95% CI)
AFP (> 5 kUI/l)	64.7% (22/34, 46.5–80.3%)	98.5% (540/548, 97.1–99.4%)	0.816 (0.735–0.898)
NC-AMRI	52.9% (18/34, 35.1–70.2%)	94.5% (518/548, 92.3–96.3%)	0.737 (0.651–0.823)
Dyn-AMRI	85.3% (29/34, 68.9–95%)	96.4% (528/548, 94.4–97.8%)	0.908 (0.847–0.969)
Complete MRI	88.2% (30/34, 72.5–96.7%)	96% (526/548, 94–97.5%)	0.921 (0.866–0.977)
NC-AMRI + AFP	88.2% (30/34, 72.5–96.7%)	93.6% (513/548, 91.2–95.5%)	0.909 (0.853–0.965)
*p* values			
AFP vs NC-AMRI + AFP	0.005	< 0.001	0.012
NC-AMRI vs Dyn-AMRI	0.005	0.086	0.001
NC-AMRI vs complete MRI	0.001	0.103	< 0.001
Dyn-AMRI vs complete MRI	0.564	0.670	0.621
NC-AMRI + AFP vs NC-AMRI	0.001	0.025	< 0.001
NC-AMRI + AFP vs Dyn-AMRI	0.706	0.014	0.979
NC-AMRI + AFP vs complete MRI	1	0.012	0.780

Abbreviations: *CI* confidence interval, *AUC* area under the curve, *AFP* alpha foetoprotein, *NC-AMRI* non-contrast abbreviated MRI, *Dyn-AMRI* dynamic abbreviated MRI

The combination of NC-AMRI + AFP in the small size HCCs subgroup significantly increased sensitivity (+ 35.3%, *p* = 0.001), from 52.9% for NC-AMRI alone to 88.2% for NC-AMRI + AFP. The sensitivity was not significantly different between NC-AMRI + AFP and complete MRI (both 88.2%, *p* = 1).

AFP alone had similar sensitivity (64.7%) to the overall population.

### False positives

On NC-AMRI, there were 30 false positive exams: 6 were negative for HCC on biopsy (cirrhotic nodules), 3 were old abscesses, 1 was a hemangioma, 1 was an inflammatory adenoma, and 19 were LI-RADS 3 without progression  ≥ 6 months. On Dyn-AMRI, there were 20 false positive exams: 4 LI-RADS 4 lesions were negative for HCC on biopsy (cirrhotic nodules), 1 LI-RADS 4 lesion was a dysplastic nodule on biopsy, 3 were hemangiomas, 3 were inflammatory adenomas, 1 was echinococcosis lesion, and 8 LI-RADS 4 lesions without washout nor progression at  ≥ 6 months. On complete MRI, there were 22 false positive exams: 9 LI-RADS 4 lesions were negative for HCC on biopsy (cirrhotic nodules), 1 LI-RADS 4 lesion was a dysplastic nodule on biopsy, 1 was an inflammatory adenoma, and 11 LI-RADS 4 lesions without washout nor progression at  ≥ 6 months.

### False negatives

**Fig. 3:**
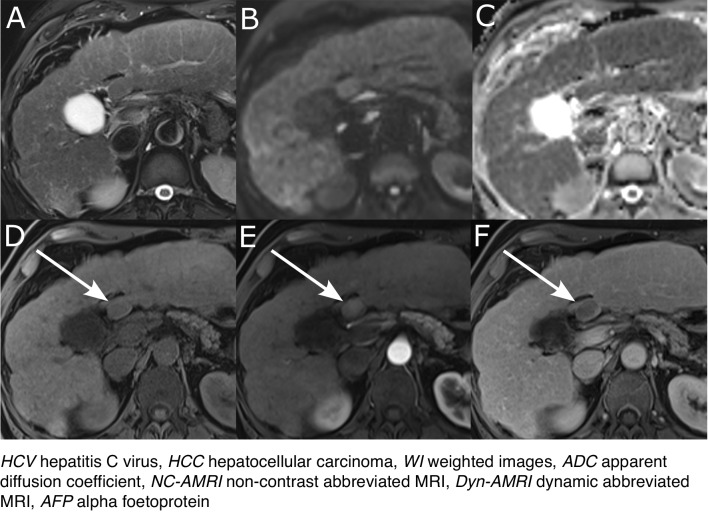
HCC patient false negative on NC-AMRI and true positive on Dyn-AMRI and NC-AMRI + AFP. A 57-year-old male patient with HCV cirrhosis and HCC. Abbreviated MRI shows a 20-mm nodule in segment IV, which is isointense on T2WI (**A**), without restricted diffusion and corresponding ADC map (**B** and **C** respectively). The same nodule is isointense on unenhanced T1WI (**D**, arrow) with homogenous arterial enhancement on arterial phase image (**E**, arrow) and washout and capsule on portal venous phase (**E**, arrow). NC-AMRI was therefore scored as negative, while Dyn-AMRI and complete MRI were scored as LI-RADS 5 (positive). NC-AMRI + AFP was considered positive, with an AFP value of 9.5 kUI/l

An example of false negative on NC-AMRI but true positive on Dyn-AMRI and NC-AMRI + AFP is shown in Fig. 3. On NC-AMRI, there were 24 false negative exams with a median size of 17 ± 5 mm (range 10–24 mm): 5 lesions were missed by the reader, 3 lesions were considered subthreshold by reader (thus not considered positive), and 16 lesions were not visible on T2WI and DWI, thus missed on NC-AMRI. On Dyn-AMRI, there were 6 false negative exams with a median size of 13 ± 2 mm (range 11–15 mm): 5 lesions were missed by the reader, 1 lesion was classified LI-RADS 3 but would have been upgraded to LI-RADS 4 based on DWI. On complete MRI, there were 5 false negative exams with a median size of 15 ± 4 mm (range 12–23 mm): 4 lesions were missed by the reader, 1 lesion was misclassified as LI-RADS 3.

## Discussion

In the present study, we compared the diagnostic performance of two AMRI sets and complete MRI in a large cohort of 631 MR examinations and 351 patients. Sensitivities of Dyn-AMRI and complete MRI (both 92.8%) were similar, higher than NC-AMRI (72.3%). The addition of AFP improved sensitivity for NC-AMRI (92.8%), however with a drop in specificity. The same was true for the small size HCCs subgroup. The difference in sensitivity between reading sets was more pronounced when considering the subgroup of small size HCCs (≤ 2 cm), where NC-AMRI evidenced a drop in sensitivity of 19.4% compared to the overall HCC size, while Dyn-AMRI and complete dropped by 7.5 and 4.6%, respectively.

Missed HCCs were small, although bigger with NC-AMRI (17 mm median size) than with Dyn-AMRI (13 mm) and complete MRI (15 mm). These results are in line with previous studies assessing AMRI for HCC detection [18, 19, 22, 24].

There were more false positive cases with NC-AMRI (*n* = 30) than with Dyn-AMRI (*n* = 20) and complete MRI (*n* = 22).

In our study, sensitivity of Dyn-AMRI with ECCA (92.8%) was in the same range than the only existing previous study (88.2%) with the same contrast agent [27]. Sensitivity was higher than with hepato-specific contrast agents (84.6%) [19], confirming the high diagnostic performance of Dyn-AMRI when performed with ECCA. Moreover, the sensitivity of Dyn-AMRI in the present study was in the higher range of previous reports on HBP-AMRI (80.6 to 92%), with similar specificity [15, 19–22, 24]. The use of Dyn-AMRI could explain why we found a statistical difference in diagnostic performance between NC-AMRI and Dyn-AMRI, on the contrary to a recent meta-analysis where no difference was found between protocols [30]. Most of the studies included in this meta-analysis used HBP-AMRI and not Dyn-AMRI.

AFP significantly improved the sensitivity of NC-AMRI. NC-AMRI + AFP showed similar AUC and sensitivity to Dyn-AMRI and complete MRI in the overall HCC size and small size HCCs. Specificity was lower in both groups (93.6% in both), however still high. These results are of interest as NC-AMRI has important advantages, compared to Dyn-AMRI: lower acquisition time (7 min vs 10 min), lower interpretation time, lower costs, and more importantly the absence of contrast injection, making it ideal for a HCC surveillance program. In the overall HCC size, NC-AMRI + AFP combination demonstrated similar sensitivity (92.8%) to the reported US + AFP combination (97% in a recent meta-analysis) [6]. When considering the small size HCCs subgroup, NC-AMRI + AFP had higher sensitivity (88.2%) than the reported sensitivity of US + AFP (63%) [6], suggesting a better diagnostic performance for HCC surveillance. Evidence is scarce regarding diagnostic performance of AMRI for small size HCCs, with two studies reporting a NC-AMRI sensitivity between 72.5% and 75% for  < 2 cm HCCs [18, 20]. Our sensitivity with NC-AMRI (52.9%) is in the lower range of these studies. The sensitivity of Dyn-AMRI remained high at 85.3%, with a drop of 7.5% compared to the overall HCC size. To note, in the recent meta-analysis of Gupta et al [30], the authors found a drop in sensitivity in small size HCCs, from 86 to 69%, but they did not perform a subgroup analysis based on the AMRI protocol. Our results suggest that the drop in sensitivity is higher with NC-AMRI than with Dyn-AMRI. In our study, thanks to the combination of NC-AMRI and AFP, the sensitivity for small size HCCs remained high (88.2%), similar to Dyn-AMRI and complete MRI. This enforces the need to take into account AFP value or contrast injection to achieve high sensitivity for HCC detection, as previously suggested [19, 20, 23, 25]. In a HCC surveillance program, a positive AFP value would lead to contrast injected MRI, without the need for NC-AMRI. However, according to our results, a negative NC-AMRI + AFP could reliably exclude HCC, making it an ideal candidate for HCC surveillance programs. Previous papers suggest that small HCCs might have lower rates of positive AFP [31]. Our results are not supporting this, as 64.7% of small HCCs were AFP positive in our cohort. However, to the best of our knowledge, no study assessed AFP values according to HCC size as a primary outcome. Therefore, further studies are needed on the subject, to clarify if small HCCs indeed have lower AFP values.

Cost-effectiveness of AMRI for HCC screening needs to be clarified before implementation in clinical practice. Parameters to take into account for cost-effectiveness are AMRI costs, diagnostic performance of AMRI, patients’ compliance, the incidence of HCC, and treatment-enhanced survival [32]. Comparison with US data is required as it is the screening reference method. So far, Goossens et al assessed risk-stratified HCC screening strategies, stratifying patients in high-, intermediate-, and low-risk for HCC. They evidenced that HBP-AMRI screening for high- and intermediate-risk patients was the highest cost-effective strategy [33]. Lima et al’s study compared costs of US, CT, MRI, and AMRI for HCC screening, and concluded that AMRI is cost-effective in a conservative scenario (52% surveillance compliance) [34]. Vietti Violi et al found similar results, with AMRI-based models compared with US showing incremental costs within currently accepted ranges [19]. These preliminary results are based on North American data, and need to be adapted to each country due to the differences in HCC screening populations and health system characteristics. In addition to high-risk populations, AMRI could be useful for patients with limited diagnostic performance of HCC screening with US, such as obesity, steatosis, or heterogeneity of liver parenchyma [35]. Overall, further studies are needed to determine which groups of patients could benefit from HCC screening using AMRI, as recently suggested [36].

The present study has limitations. First, the retrospective design did not allow comparison with US, which is the reference method for HCC surveillance. However, it allowed a large population series. Second, our cohort is a selected population of individual screening rather than a true screening population, as our prevalence of HCC (23.6%) is higher than the expected 3–4% of a surveillance population [37]. The diagnostic performance of our AMRI sets could therefore be overestimated. However, this allowed sensitivity analysis, which would be limited in prospective studies due to the low rate of positive cases. Third, unlike during a true screening program, the readers did not have access to previous examinations, thus not allowing the assessment of threshold growth. Fourth, the added value of AFP to NC-AMRI might be overestimated, highlighting the need of prospective validation.

In this large retrospective study, sensitivity of Dyn-AMRI (92.8%) outperformed NC-AMRI (72.3%) for HCC detection in patients at risk, and had similar sensitivity compared to complete MRI (92.8%). However, the combination of NC-AMRI + AFP provided similar sensitivity to Dyn-AMRI and complete MRI, questioning the need of contrast injection when considering HCC surveillance with AMRI. In the subgroup of small size HCCs (≤ 2 cm), sensitivity remained high for Dyn-AMRI (85.3%) and NC-AMRI + AFP (88.2%), while sensitivity dropped for NC-AMRI alone (52.9%).

Further studies are needed, especially to prospectively investigate US, NC-AMRI + AFP, and Dyn-AMRI and to perform cost-effectiveness analysis in a clinical scenario integrating each HCC population characteristics.

### Supplementary Information

Below is the link to the electronic supplementary material.

Supplementary file1 (PDF 15 KB)

## Abbreviations

AMRI: Abbreviated magnetic resonance imaging
AUC: Area under the curve
CI: Confidence interval
Dyn-AMRI: Dynamic AMRI
ECCA: Extracellular contrast agent
HBP-AMRI: Hepato-biliary phase contrast AMRI
HCC: Hepatocellular carcinoma
LI-RADS: Liver Imaging Reporting and Data System
NC-AMRI: Non-contrast AMRI
SD: Standard deviation
US: Ultrasound
WI: Weighted imaging

## Declarations

### Guarantor

The scientific guarantor of this publication is Naik Vietti Violi.

### Statistics and biometry

Mario Jreige and Yasser Aleman kindly provided statistical advice for this manuscript.

### Study subjects or cohorts overlap

No study subjects or cohorts have been previously reported.

### Methodology

• retrospective

• diagnostic study

• performed at one institution
