# Supplementary material for: The combination of non-contrast abbreviated MRI and alpha foetoprotein has high performance for hepatocellular carcinoma screening
Source: Eur Radiol. 2023 Jul 18;33(10):6929–38. doi: 10.1007/s00330-023-09906-4 (PMC10511584; doi:10.1007/s00330-023-09906-4)
Supplement: Supplementary file 1 — Supplementary file1 (PDF 15 KB) [file 330_2023_9906_MOESM1_ESM.pdf]

The combination of non-contrast abbreviated MRI and alpha foetoprotein has high performance for hepatocellular carcinoma screening

Electronic Supplementary Material

MRI protocol

Examinations were performed with a surface body array coil. Our clinical liver MRI protocol included the following sequences: breath-hold axial and coronal T2 weighted imaging (WI) half-Fourier acquisition single-shot turbo spin-echo (T2WI HASTE), a respiratory-triggered fat-suppressed turbo spin-echo T2WI, a free breathing fat-suppressed single-shot echoplanar diffusion weighted imaging (DWI) with tridirectional diffusion gradients using b-values of 50, 400, 800s/mm^2 and a breath-hold axial T1WI using a fat-suppressed three-dimensional spoiled gradient-echo sequence, volumetric interpolated (VIBE) obtained before and after contrast injection. An intravenous injection of 0.1 mmol/kg of gadoterate meglumine (Dotarem; Guerbet) at a rate of 3 ml/s was then performed, followed by a bolus of 20 ml of saline solution at a rate of 2 ml/s. Triple arterial phase images were acquired after a delay of 20 seconds with a Caipirinha (controlled aliasing in parallel imaging results in higher acceleration) Dixon TWIST (time-resolved angiography with stochastic trajectories) volume interpolated breath-hold examination sequence (VIBE). Portal venous (axial), equilibrium (axial) and delayed (axial and coronal) phases were acquired, at 70 sec, 3 min and 5 min, respectively.
